# The mTORC1 Signaling Support Cellular Metabolism to Dictate Decidual NK Cells Function in Early Pregnancy

**DOI:** 10.3389/fimmu.2022.771732

**Published:** 2022-03-10

**Authors:** Song Yan, Jie Dong, Chenxi Qian, Shuqiang Chen, Qian Xu, Hui Lei, Xiaohong Wang

**Affiliations:** Department of Gynecology and Obstetrics, Tangdu Hospital, Air Force Medical University, Xi’an, China

**Keywords:** decidual NK cells, metabolism, mTORC1, cytokines, cytotoxicity, RPL

## Abstract

Cellular metabolism plays an important role in regulating both human and murine NK cell functions. However, it remains unclear whether cellular metabolic process impacts on the function of decidual NK cells (dNK), essential tissue-resident immune cells maintaining the homeostasis of maternal-fetal interface. Remarkably, we found that glycolysis blockage enhances dNK VEGF-A production but restrains its proliferation. Furthermore, levels of IFN-γ and TNF-α secreted by dNK get decreased when glycolysis or oxidative phosphorylation (OXPHOS) is inhibited. Additionally, glycolysis, OXPHOS, and fatty acid oxidation disruption has little effects on the secretion and the CD107a-dependent degranulation of dNK. Mechanistically, we discovered that the mammalian target of rapamycin complex 1 (mTORC1) signaling inhibition leads to decreased glycolysis and OXPHOS in dNK. These limited metabolic processes are associated with attenuated dNK functions, which include restricted production of cytokines including IFN-γ and TNF-α, diminished CD107a-dependent degranulation, and restrained dNK proliferation. Finally, we reported that the protein levels of several glycolysis-associated enzymes are altered and the mTORC1 activity is significantly lower in the decidua of women with recurrent pregnancy loss (RPL) compared with normal pregnancy, which might give new insights about the pathogenesis of RPL. Collectively, our data demonstrate that glucose metabolism and mTORC1 signaling support dNK functions in early pregnancy.

## Introduction

Decidual natural killer cells (dNK) represent the largest population of decidua-resident immune cells, comprising about 50–70% of the maternal immune cells across early pregnancy (1). Studies have revealed that dNK are phenotypically and functionally distinct from peripheral NK cells (pNK) and exert multiple functions to maintain homeostasis of the decidual microenvironment. dNK can directly promote vascular angiogenesis by producing vascular endothelial growth factor (VEGF) and placental growth factor (PLGF) (2, 3). In addition, IFN-γ produced by dNK is involved in promoting spiral artery remodeling (4) and inhibiting inflammatory T helper 17 (TH17) cells response to maintain immune tolerance (4). dNK also can acquire cytotoxic abilities when infection occurs (5–7). Furthermore, dysfunction of dNK have been associated with several pregnancy-related diseases, including recurrent pregnancy loss (RPL), pre-eclampsia, and fetal growth restriction (4, 8, 9). Previous studies indicated that dNK in the former disorders showed altered cytokines/chemokines and growth factor producing abilities, such as VEGF-A, IFN-γ, TNF-α, pleiotrophin, and osteoglycin (8, 10, 11). Nevertheless, the underlying mechanisms determining dNK function under physiological and pathological conditions remain to be elucidated.

Emerging evidence has highlighted the role of NK cell metabolic process in facilitating potent NK cell effector functions (12, 13). Glucose is the primary cellular fuel, and its metabolism generates both energy and biomolecules. Glycolysis converts glucose into pyruvate, which then undergoes two major fates. One is metabolized to lactate to rapidly produce 2 mol ATP. This process also generates biosynthetic precursors helpful to the survival of rapidly proliferating cells and immune cells. The other fate is to enter the mitochondria, where pyruvate is further metabolized by the tricarboxylic acid (TCA) cycle. Consequently, outputs from the TCA fuel oxidative phosphorylation (OXPHOS) to produce ATP in a more efficient way. Additionally, acetyl-CoA transformed from fatty acid oxidation (FAO) could also enter the TCA cycle (12, 14). It has been reported that in acute immune response, mouse NK cells remain low levels of glycolysis and OXPHOS, similar to that of resting state. However, these low-rate metabolism is necessary to the acute NK cell immune response, as either inhibiting glycolysis or OXPHOS can impair IFN-γ production (15). Furthermore, NK cells also function over prolonged periods, and play important roles in the adaptive immune response. Significantly elevated glycolysis and OXPHOS are observed in sustained activation of murine NK cells or human pNK, these metabolic changes are required for NK cell secretion and cytotoxicity (12, 16). Additionally, glucose-driven glycolysis and OXPHOS have been shown to drive pNK cell anti-tumor and anti-viral effector functions (17, 18), and elevated glycolysis rather than OXPHOS is associated with NK cell education (14, 19–21). Although FAO can produce acetyl-CoA to enter TCA cycle, limiting FAO has no effect on the function of NK cells producing IFN-γ (12, 22).

The mammalian target of rapamycin complex 1 (mTORC1) is a central molecule that integrates metabolic and functional changes in both murine and human NK cells (14, 15). For instance, in activated murine NK cells, enhanced glycolysis is a prerequisite for IFN-γ production and increased granzyme B expression and the strengthened mTORC1 activity following NK cells activation is required to maintain this altered metabolic profile (12). Also, human pNK activation either by cytokines stimulation or NK cell receptor ligation is dependent on an increased mTORC1 activity to supply pNK with elevated glycolysis and OXPHOS, consequently, this metabolic change prepares pNK immune response in an efficient way (16, 18, 23). However, the knowledge about the metabolic profile of dNK and its impact on dNK function and the role of mTORC1 in dNK metabolism and function is still scarce.

As tissue-resident NK cells, dNK face different nutritional conditions and metabolic requirements from pNK. The microenvironment of pNK is rich in glucose and oxygen, since blood glucose and oxygen levels are tightly regulated in healthy individuals. On the contrary, glucose and oxygen in the decidua need to be constantly replenished *via* the bloodstream. This process can be influenced by blood circulation and inflammation, which may result in local differences in nutrient levels (12, 24). Therefore, this distinct microenvironment might lead to divergent NK cells metabolism profiles, which account for the phenotype and function difference between dNK and pNK. In addition, the hypoxia environment and the inflammation in RPL have been reported (25–28), which possibly affect both dNK metabolism and function consequently.

This study aims to investigate dNK metabolism characteristics and study the potential role of the mTORC1 signaling and metabolic processes in dNK function under physiological and pathological conditions.

## Materials and Methods

### Tissue Samples and Blood

Decidual tissues of healthy early pregnancies (n = 29) were obtained from elective pregnancy terminations (age ranged from 20 to 35, 28 ± 4.5 years; gestational age at sampling, 45 ± 5 days; BMI, 21.4 ± 3.1, mean ± standard variation (SD)). Peripheral blood mononuclear cells (PBMCs), including peripheral NK cells (pNK), were isolated from healthy nonpregnant volunteer blood donors with no known significant health problems evaluated by a physical exam within three months (n = 5, age ranged from 25 to 32, 28 ± 2.7 years; BMI, 21.3 ± 1.4, mean ± SD). Decidua of abnormal pregnancies (n = 19) was obtained from patients with recurrent pregnancy loss (RPL) (age ranged from 26 to 35, 30 ± 2 years; gestational age at sampling, 60 ± 5 days; BMI, 22.3 ± 2.4, mean ± SD). Recurrent pregnancy loss was defined as two or more failed clinical pregnancies, which are verified by ultrasonography or histopathology (28). Patients with the following features were excluded: (1) anatomic abnormalities on pelvic examination or ultrasound, (2) chromosomal abnormalities, (3) symptoms of endocrine or metabolic diseases. After the individuals signed the informed consent, the samples were collected from patients and our laboratory colleagues at Tangdu Hospital, Air Force Medical University. This study was approved by the Ethics Committee of Tangdu Hospital, Air Force Medical University. In addition, 15 decidual samples of those 29 healthy early pregnancy patients described above and 19 RPL patients were collected for Western blotting. Tissues for Western blot analysis were cleaned and then quickly frozen in liquid nitrogen and stored at -80°C before use.

### Cell Preparation and Purification

To isolate decidual immune cells (DICs), decidual tissue was washed, minced, and digested with 0.1 mg/mL collagenase IV (Gibco) and 0.01 mg/mL DNase I (Sigma) in RPMI medium1640 (Gibco) shaking in a water bath for 1 h at 37°C. Released lymphocytes were filtered through nylon mesh. DICs were isolated by Ficoll (TBD Cat# LDS1077) density gradient centrifugation (30 min at 800 × g). Purification of dNK from DICs requires adherent culture overnight to remove stromal cells. Decidual NK cells were further purified by negative selection using a magnetic-activated cell sorter (MACS) kit (Miltenyi Biotec, #130-092-657). For all dNK, >90% purity was obtained (**Supplementary Figure 1A**).

Similarly, PBMCs were isolated using Ficoll density gradient centrifugation (30 min at 800 × g). Peripheral NK cells (pNK) were further purified by the same MACS negative selection kit (Miltenyi Biotec). The purity of the resulting pNK population was greater than 90% (**Supplementary Figure 1B**).

### Cell Culture and Treatment

DICs, dNK, and pNK were isolated and routinely cultured in RPMI medium1640 (Gibco) supplemented with 10% FBS (Corning), 1% penicillin/streptomycin, and 20 ng/ml IL-15 (PeproTech) in 5% CO2 at 37 °C. In some experiments, DICs were cultured under the following two conditions (29, 30): (i) IL-15 (20 ng/mL); IL-15 is routinely added in the culture of dNK as a survival factor; (ii) a cytokine cocktail (IL-12, 10 ng/ml; IL-15, 10 ng/ml; IL-18, 50 ng/ml; all from Peprotech); This culture condition mimic a pro-inflammation state in RPL to fully activate dNK (30). Work concentrations of inhibitors were referred to published literature on pNK. To inhibit different cellular metabolic processes, DICs were treated with 2-DG (50 mM; MCE) to inhibit glycolysis, oligomycin (1 μM;MCE) plus the combination of rotenone (500 nM;MCE) and antimycin A (500 nM; Sigma) to inhibit OXPHOS, and etomoxir (10 μM; MCE) to inhibit fatty acid oxidation (17, 18, 21, 31). Rapamycin (20 nM; MCE) was added to suppress mTORC1 activity (32).

### Seahorse Analysis

We used a Seahorse XFe24 analyzer (Seahorse Bioscience) to real-time analyze the extracellular acidification rate (ECAR) and oxygen consumption rate (OCR) of NK cells cultured under various conditions. In brief, purified dNK and pNK were cultured for 24 h supplemented with IL-15 as a survival factor, then they were adhered to 24-well plate using Cell-Taq (BD Pharmingen) at 300,000 cells/well. During analysis,10 mM glucose, 0.5 µM oligomycin, and 100 mM 2-deoxyglucose (2-DG) were injected sequentially, being used to determine non-glycolytic acidification, glycolysis, glycolytic capacity, and glycolytic reserve. Consecutive additions of oligomycin (1 µM), FCCP (1 µM, Sigma), and rotenone (500 nM) plus antimycin A (500 nM) allowed to obtain with accuracy the oxygen consumption due to basal respiration, maximal respiration, ATP production and non-mitochondrial respiration.

### Flow Cytometry (FCM)

The antibodies used in this study for the flow cytometric analysis are listed in **Supplementary Table I**.

For surface staining, cells were stained for 30 min on ice in phosphate buffer saline (PBS) with 1% fetal bovine serum (FBS) after 10 min incubation with Fc-Block (Biolegend). For intracellular staining, cells were cultured overnight under two conditions described above, then brefeldin A (BFA) and monensin (Biolegend) were added for another 4 h at 37°C. Cells were harvested and stained for surface markers (CD45, CD3, and CD56). Subsequently, these cells were fixed, permeabilized (Fix/Perm Buffer Set, Biolegend) and stained with anti-human VEGF, IFNG-γ, and TNF-α to assess intracellular cytokines expression. In addition, Ki-67 was stained by the Foxp3/Transcription Factor Staining Buffer Set (eBioscience) under manufacturer’s instructions. Antibodies used were conjugated anti-CD3, anti-CD56, and anti-Ki-67. For all experiments, matched immunoglobulin G (IgG) antibody was used as isotype controls. Flow cytometric data were acquired with a BD FACS Aria II flow cytometer (BD Biosciences). Offline data analyses were performed by FlowJo V10 (TreeStar) or Kaluza 1.3 (Beckman Coulter) software.

### dNK Degranulation Assays

For degranulation assays, DICs were cultured overnight in complete culture medium with 2.5 ng/ml IL-15. The DICs (effector) were counted and cocultured with and without 2.5 ng/ml PMA in combination with 0.1 mg/ml ionomycin (PMA/I) or K562 (target) in a 1:1 E:T ratio in 96-well plates for 2 h. At the same time, 250 ng/mL CD107a-APC antibody was added to all cultures. After that, cells were collected and fixed for 10 min with 1% paraformaldehyde and subsequently stained with CD3-FITC and CD56-PE for FCM analysis.

### CFSE Assay

DICs were labeled with 5 μM CFSE (Biolegend) for 10 min at 37°C. The labeled DICs were cultured in complete medium with IL-15 (20 ng/ml) under different treatments as indicated for 6 days. Cells were then harvested, CFSE MFI of CD3^-^CD56^+^ NK cells were analyzed. Experiments were replicated at least three times, and the proliferation ability of dNK was compared by analyzing the relative intensity of CFSE MFI.

### Western Blot

Briefly, tissues were homogenized and lysed with the radioimmunoprecipitation assay buffer (Thermo) containing mixed inhibitors of protease (Roche) and phosphatase (MCE). Twenty micrograms of the protein were subjected to 4-20% precast gel (GenScript, China) for separation and then transferred to PVDF membranes. The membranes were blocked with 5% BSA in TBST for 1 h and incubated overnight at 4 °C with primary antibodies (1:1000 for HK-I, HK-II, PFKP, PKM2, PKM1/2, PDG, LDHA, p-S6, t-S6, p-4EBP1 and t-4EBP1; and 1:5000 for GAPDH and β-actin). Anti-β-actin antibody was used as the loading control. All the antibodies for Western blotting analysis were purchased from Cell Signaling Technology. The blots were then incubated with peroxidase-conjugated secondary antibodies, and the chemiluminescence signal was detected using a ChemiDocTM Touch Imaging System (Bio-Rad).

### Statistical Analysis

All experiments were performed at least three times, and data were presented in the form of means ± standard error of mean (SEM). GraphPad Prism 7.00 (GraphPad Software) was used for statistical analysis. Normality of data was tested, and if a nonnormal distribution was found, the nonparametric test was used. A one-way ANOVA test compared more than two groups throughout with a Dunnett post-test for multiple comparisons. A two-tailed Student’s t-test for analysis was used when there were two groups. For comparison of relative values, column statistics were used to calculate *P*-value with the theoretical value of 100. A *P* < 0.05 was considered as statistically significant. In all figures, * *P* < 0.05, ** *P* < 0.01, *** *P* < 0.001; and ^#^ *P* < 0.0001.

## Results

### Glucose Metabolism Characteristics of Decidual Natural Killer Cells

**Figure 1:**
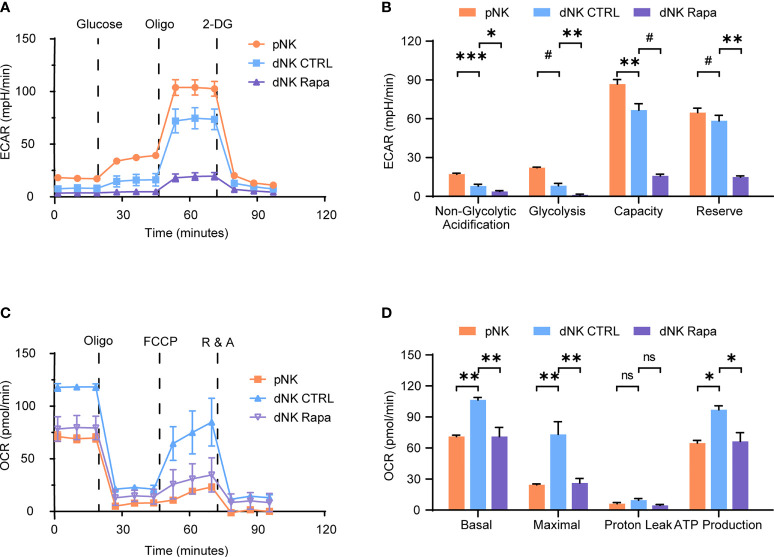
Characteristics of decidual natural killer cells glucose metabolism. **(A–D)** Analyses of extracellular acidification rate (ECAR) and oxygen consumption rate (OCR) in purified human peripheral NK cells (pNK) and decidual NK cells (dNK) after cultured for 24 h supplemented with IL-15 as a survival factor. ECAR of NK cells with the sequential addition of glucose (10 mM), the mitochondrial ATP synthase inhibitor oligomycin (oligo, 0.5 µM), and the glucose analog 2-deoxyglucose (2-DG, 100 mM) were assessed in real time **(A)**. Real-time changes of OCR in NK cells with successive addition of oligomycin (oligo, 1 µM), the mitochondrial uncoupler FCCP (1 µM), and inhibitors of mitochondrial electron transport chain complex I and III, rotenone (500 nM) and antimycin A (500 nM) (R&A), were measured **(C)**. Also, rapamycin (Rapa, 20 nM) was used to treat dNK to evaluate mTORC1 activity on its metabolism **(A–D)**. (n = 4-5 for **(A–D)**, one-way ANOVA with a Dunnett post-test). ns, not significant; *P < 0.05, **P < 0.01, ***P < 0.001; and ^#^ *P* < 0.0001.

To characterize the decidual natural killer cells (dNK) glucose metabolic processes and compare with that of peripheral natural killer cells (pNK), a Seahorse XFe24 analyzer was employed. The extracellular acidification rate (ECAR) is mainly determined by the rate of glycolysis. In contrast, the oxygen consumption rate (OCR) is an indicator of mitochondrial respiration, also known as oxidative phosphorylation (OXPHOS). The results showed that dNK had decreased glycolysis, lower glycolytic capacity, and less glycolytic reserve than pNK (**Figures 1A, B**). Reciprocally, dNK exhibited increased basal respiration, greater maximal respiratory capacity, and higher ATP production (**Figures 1C, D**).

The mammalian target of rapamycin complex 1 (mTORC1) is a crucial regulator of cellular metabolism and has essential roles in controlling immune cell function. Therefore, the effect of mTORC1 on dNK glucose metabolism was investigated. Metabolic analysis of dNK in the presence of rapamycin (Rapa) revealed that mTORC1 activity inhibition led to both reduced OCR and ECAR profiles (**Figures 1A–D**). Together, these data suggest a discrepancy found in glycolysis and OXPHOS between dNK and pNK. Moreover, the mTORC1 activity support dNK glycolysis and OXPHOS.

### Role of Metabolism in dNK Cytokines Secretion

**Figure 2:**
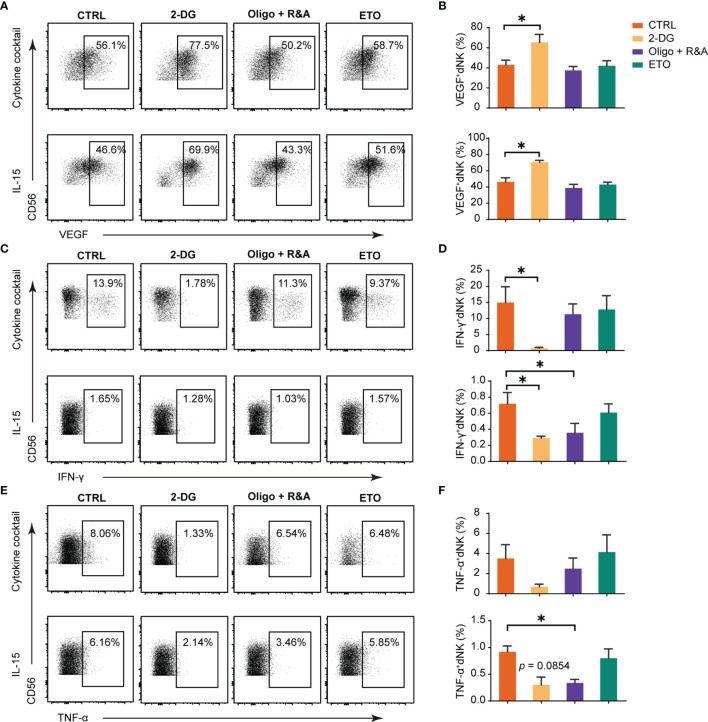
Glucose metabolism as a determinant factor in dNK cytokines production. Representative dot plots of VEGF-A, IFN-γ, and TNF-α in decidual NK cells (dNK) (gated as CD45^+^CD3^-^CD56^+^). Decidual immune cells (DICs) were cultured under the following two conditions (29, 30): (i) IL-15 (20 ng/mL); (ii) a cytokine cocktail (IL-12, 10 ng/ml; IL-15, 10 ng/ml; IL-18, 50 ng/ml);. These cells were cultured added with 2-DG (50 mM) to inhibit glycolysis, oligomycin (1 μM) plus the combination of rotenone (500 nM) and antimycin A (500 nM; Oligo+R&A) to inhibit OXPHOS, and etomoxir (ETO, 10 μM) to inhibit fatty acid oxidation (FAO), respectively. Then, cells were collected for intracellular cytokine staining for VEGF-A **(A, B)**, IFN-γ **(C, D)**, and TNF-α **(E, F)**. (n = 5 for all experiments, paired t-test). *P < 0.05.

Cytokine secretion is a crucial function of dNK (10). We next utilized a series of well-established pharmacological inhibitors to explore how different cellular metabolic processes potentially affect dNK producing cytokines. The results showed that dNK secreted significantly higher level of VEGF-A both in cocktail and single IL-15 culture conditions after the treatment with 2-DG to inhibit glycolysis, (**Figures 2A, B**). However, minimal or no effects were found in OXPHOS blocking or fatty acid oxidation (FAO) disruption groups (**Figures 2A, B**). Although the data were not significant in some culture conditions, the inhibition of glycolysis or OXPHOS could repress the levels of IFN-γ or TNF-α. In contrast, the fatty acid oxidation (FAO) disruption showed minor effects on IFN-γ and TNF-α production (**Figures 2C–F**).

Overall, glycolysis inhibition could enhance dNK VEGF-A production, while glycolysis or OXPHOS blocking could reduce the levels of IFN-γ or TNF-α. Contrastingly, FAO metabolism blockage has little effects on these cytokines production.

### dNK Cytotoxicity Is Not Influenced by Short-Term Metabolism Alteration

**Figure 3:**
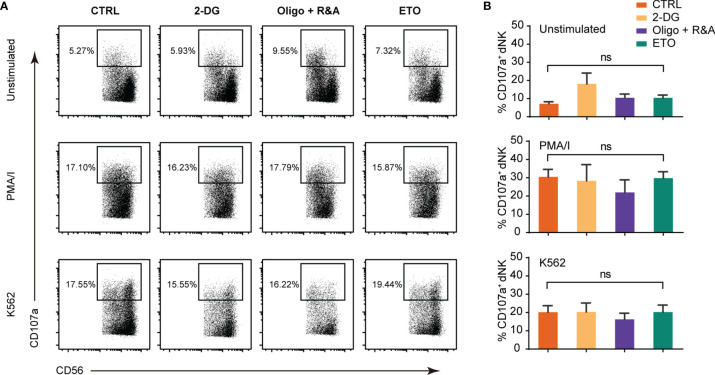
Metabolism blockage has minimal effects on dNK cytotoxicity abilities. **(A)** Representative FCM plots of CD107a staining of gated CD3^-^CD56^+^ NK cells in decidual immune cells (DICs) cultured alone (top panels), with PMA/I (middle panels, 2.5 ng/ml PMA in combination with 0.1 mg/ml ionomycin), and with K562 (bottom panels). **(B)** Percentage of CD107a+ NK cells upon being cultured alone (upper), with PMA/I (middle), and K562 (lower). All cells were cultured in the presence of 2.5 ng/ml IL-15 for 2 h added with CD107a antibody. (n = 4–5, nonparametric one-way ANOVA). ns, not significant.

dNK cytotoxicity was studied by measuring CD107a-dependent degranulation, a marker associated with NK cell cytotoxicity in response to MHC-negative K562 targets cells and PMA/ionomycin (PMA/I) stimulation. As shown in **Figures 3A, B**, the response of the first-trimester dNK to K562 and PMA/I was relatively low, in accordance with a weaker cytotoxicity phenotype compared with pNK (7, 33). Furthermore, no significant change in the dNK CD107a-dependent degranulation was observed upon glycolysis, OXPHOS, and FAO was blocking. These results demonstrate that short-term metabolism alteration has minimal or no effects on dNK cytotoxicity.

### Glycolysis Is Indispensable for dNK Proliferation

**Figure 4:**
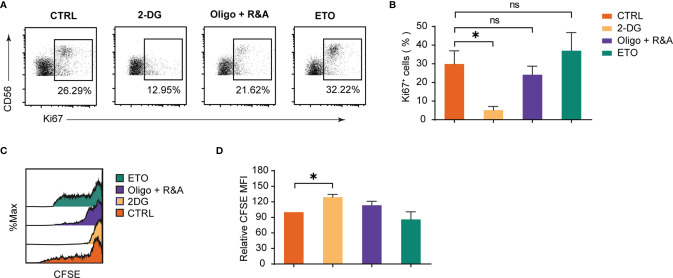
Glycolysis underpins dNK proliferation. **(A, B)** Ki-67 staining on decidual immune cells (DICs) after treatment with various metabolism inhibitors for 48h. DICs were routinely cultured in full culture medium supplemented with 20 ng/ml IL-15. After treatment, Ki-67 positive dNK (CD3^-^CD56^+^ cells) were analyzed by FCM (n = 5-6, paired t test). **(C, D)** Representative histogram shows CFSE dilution of dNK (CD3^-^CD56^+^ cells). The summary graph shows the relative CFSE intensity of dNK (n = 5, One sample t-test). ns, not significant; *P < 0.05.

The metabolism influence on dNK proliferation was studied by the Ki-67 staining (**Figures 4A, B**) and the CFSE dilution method (**Figures 4C, D**). The results demonstrated that only attenuated glycolysis blocked by 2-DG significantly inhibited Ki-67 positive dNK percentages. Furthermore, an elevated dNK CFSE MFI was observed following glycolysis inhibition. At the same time, other pharmacological inhibitors did not induce a dNK CFSE MFI change, which was consistent with the results of Ki-67 staining. Altogether, these data reveal that glycolysis is the most critical mechanism for dNK proliferation among diverse metabolic processes.

### mTORC1 Regulate dNK Secretion and Cytotoxicity Abilities

**Figure 5:**
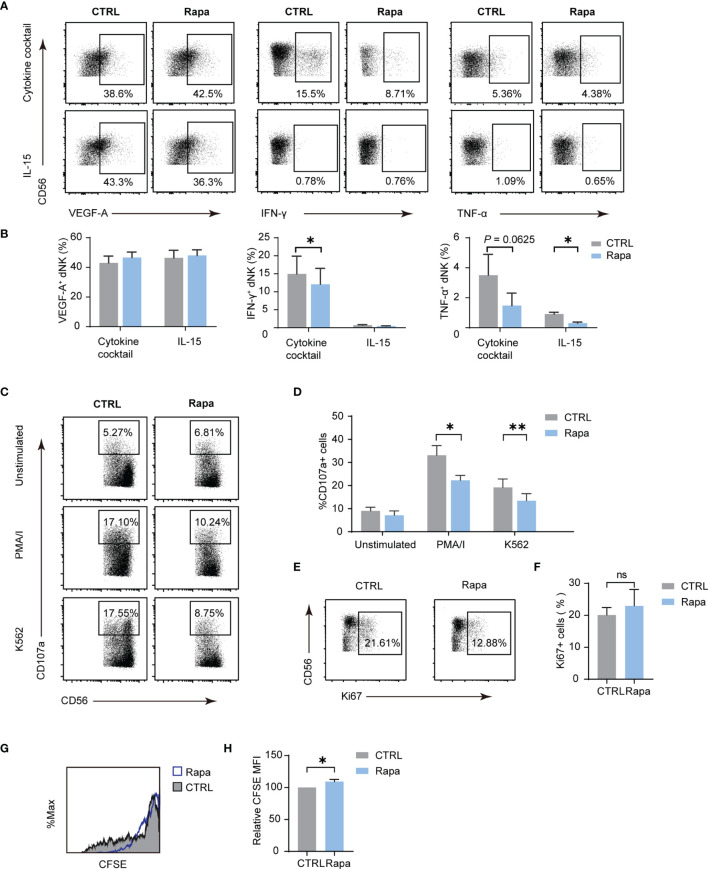
The mTORC1 modulate dNK secretion, cytotoxicity abilities, and proliferation. Decidual immune cells (DICs) were cultured added with rapamycin (Rapa, 20 nM) to inhibit mTORC1 activity. As described above, decidual NK cells (dNK) cytokines production, cytotoxicity abilities, and proliferation were examined. **(A, B)** Rapa treatment lowered the frequency of IFN-γ and TNF-α, even though VEGF-A production was not altered (n = 4-5, paired t-test). **(C, D)** Diminished mTORC1 activity reduced CD107a-dependent degranulation (n = 5, paired t-test). **(E–H)** Ki-67 positive dNK percentage was not influenced by Rapa (n = 5, paired t-test); CFSE dilution assay showed a little increase in dNK CFSE MFI compared with controls (n = 4, paired t-test). ns, not significant; *P < 0.05; **P < 0.01.

The mammalian target of rapamycin complex 1 (mTORC1) is a critical regulator of NK cell metabolism and function, and studies have shown that pNK functions are intensively dictated by metabolism (12, 14). The results presented indicate that mTORC1 is also implicated in dNK metabolism. To investigate whether dNK function is associated with mTORC1 activity, a frequently used mTORC1 inhibitor, namely Rapa, was employed. As shown in **Figures 5A, B**, the use of Rapa led to a decrease in the percentage of IFN-γ and TNF-α, while VEGF-A production was not altered. For dNK cytotoxicity, mTORC1 activity decline resulted in diminished CD107a-dependent degranulation (**Figures 5C, D**). Although Ki-67 positive dNK percentage was not influenced by Rapa, CFSE dilution assay showed a significant decrease in dNK division (**Figures 5E–H**). Collectively, these results underscore that mTORC1 activity modulates dNK cytokines production, cytotoxic reaction, and cell proliferation.

### Dysregulation of Decidual Glycolysis and mTORC1 Activity Is Associated With RPL

**Figure 6:**
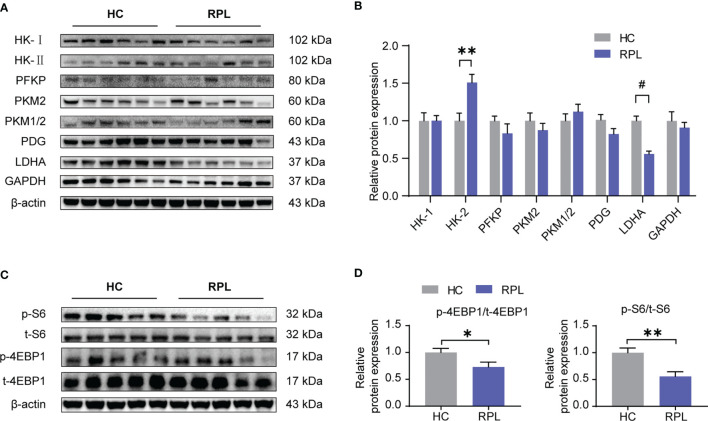
Expression of glycolysis enzymes was altered and the mTORC1 signaling pathway was downregulated in RPL decidua. **(A, B)** Western blotting and quantitative results of glycolysis-associated enzymes and GAPDH in the decidua of women with normal early pregnancies (n = 15) and those with recurrent pregnancy loss (RPL) (n = 19). HC, health control; RPL, recurrent pregnancy loss (unpaired t-test). **(C, D)** Western blot analysis the mTORC1 activity in some samples described as above. Also, phosphorylated S6 ribosomal protein(p-S6)/total S6 and phosphorylated eukaryotic translation initiation factor 4E-binding protein 1 (p-4EBP1)/total 4EBP1 expression were analyzed by densitometry method (n = 6 for both groups, unpaired t test). *P < 0.05, **P < 0.01, and ^#^ *P* < 0.0001.

Considering mTORC1 regulates dNK functions and this effect is dependent on cellular metabolism, we next investigated if glycolysis and mTORC1 activity is altered in the RPL decidua. In the decidua of RPL, of these enzymes associated with glycolysis, it was found that the protein level of HK-II and LDHA was significantly changed (**Figures 6A, B**), while the others did not change. We also found that the levels of phosphorylated S6 ribosomal protein (p-S6) and phosphorylated eukaryotic translation initiation factor 4E-binding protein 1 (p-4EBP1), readouts of mTORC1 signaling, were significantly lower in RPL tissues compared with normal pregnancy samples (**Figures 6C, D**). Thus, during RPL, aberrant glycolysis, the decreased mTORC1 activity, and the impairment of dNK are present in the decidual tissues.

## Discussion

dNK constitute a unique tissue-resident NK cell subset that reside at the maternal-fetal interface. Previous studies have revealed that dNK play a critical role in early placenta development, promoting fetal growth, maintaining immunologic tolerance, and anti-viral immunity (10, 34, 35). Dysregulation of dNK is implicated in pregnancy-related diseases, including recurrent pregnancy loss (RPL), pre-eclampsia, and fetal growth restriction (4). Nonetheless, the regulatory mechanisms determining dNK function under physiological and pathological conditions remains unclear. In this context, the present study is the first to characterize the metabolism of human dNK and identify that these versatile NK cells have distinct metabolic phenotypes. Compared with pNK, dNK showed a higher OXPHOS but a lower glycolysis metabolism profile. Remarkably, this study found that dNK metabolic process underpinned dNK functions: glycolysis blockage would enhance dNK VEGF-A production while restraining cellular proliferation. In addition, levels of IFN-γ and TNF-α secreted by dNK got decreased upon glycolysis and/or OXPHOS was restricted. Moreover, this study demonstrated that mTORC1, the master sensor of essential nutrients, supports dNK metabolism and impacts on dNK function. Detailed metabolic analysis showed that mTORC1 maintains glycolysis and OXPHOS in dNK, which was associated with dNK function, including production of cytokines like IFN-γ and TNF-α, CD107a-dependent degranulation, and dNK proliferation. Interestingly, in the decidua of RPL, disorganized glycolysis and decreased mTORC1 activity were observed. Thus, this study represents a forward step, demonstrating that altered decidual metabolism and mTORC1 might be associated with dNK dysregulation in RPL, which might shed light on the pathogenesis of RPL.

Cellular metabolism is primarily determined by its microenvironment, including but not limited to nutrient and oxygen supply. Indeed, metabolic processes dictate cell functional fate to some extent (13). For example, both tumor-associated NK cells and dNK are tissue-resident cells, live under oxygen deprivation and in an immunosuppressive microenvironment (36). These cells exhibit low or even non-cytotoxic properties, but potent proangiogenic effects (35, 36). TGF-β was presented in the tumor microenvironment, and it can also be produced by decidual stromal cells at the maternal-fetal interface (36). Cerdeira *et al.* found that TGF-β combined with hypoxia was sufficient to convert pNK into regulatory NK cells that secrete high levels of VEGF-A but have poor cytotoxicity (37). Additionally, TGF-β could attenuate NK cell cytotoxic functions, which depends on suppressing glucose-driven glycolysis and OXPHOS (38, 39), implying effects that cellular metabolism impacts the functional property of NK cells. In this study, the inhibition of glycolysis improves VEGF-A production. This result is in consistent with the notion that dNK’s VEGF-A secreting ability is more potent than that of pNK (36, 37), as a weaker glycolysis and higher OXPHOS is observed in dNK compared with pNK in our experiment. Similarly, in the lung tumor environment, glycolysis inhibition caused by upregulated fructose-1,6-bisphosphatase (FBP1) expression, a rate-limiting enzyme in gluconeogenesis, is observed in this tumor-associated proangiogenic NK cells (40). These evidences suggest that NK cell producing VEGF-A is regulated by glycolysis. Therefore, future studies are warranted to investigate how dNK producing VEGF-A is regulated by glycolysis. IFN-γ and TNF-α production by dNK like pNK might rely on elevated glycolysis and OXPHOS (41). Curiously, any effects of different metabolic process alteration on dNK CD107a-dependent degranulation were found, indicating that other mechanisms may exist to determine dNK cytotoxicity independence of cellular metabolism.

The mTORC1 signal pathway is the most studied mechanism to regulate NK cell metabolism and functions. Despite the majority of studies are focused on effects of mTORC1 on the metabolism and function of pNK, there is a paucity of literature exploring its potential role in tissue-resident NK cells, including dNK. Previous studies have revealed that IL-15 controls differentiation, homeostasis, and activation of NK cells, in which mTORC1 signaling play crucial roles. Furthermore, IL-15 is present at the decidua physiologically, and its production is hormonally controlled, especially by progesterone (42, 43). In this study, mTORC1 was reported to support dNK metabolism and immune response. The mTORC1 activity attenuation causes diminished glycolysis and OXPHOS in dNK, accompanied by a weaker dNK function, such as decreased cytokines secretion, including IFN-γ and TNF-α, diminished CD107a-dependent degranulation, and lowered dNK proliferation. This regulatory effect agrees with what was described in the case of pNK (20, 44). Consequently, it is likely that the mTORC1 signaling pathway support cellular metabolism to dictate dNK function at the human maternal-fetal interface.

Previous reports have showed an over-inflammation state in the decidua of RPL (29, 35). Besides, increased HIF1α expression and micro-vessel density have been documented in peri-implantation endometrium in women with a history of RPL (27, 28). These features may change the microenvironment where dNK inhabit. Consequently, dNK cellular metabolism and functions are impaired, which might participate in the pathogenesis of RPL. Indeed, recent studies have revealed that glycolysis disturbance occurs at the decidua of RPL, and attenuated ECAR and OCR in RPL dNK compared with healthy controls has also been reported (45, 46). In this study, protein levels of HK-II and LDHA were found to be altered, and a decreased mTORC1 activity was observed by Western blot in the RPL decidual samples analyzed. Therefore, aberrant glycolysis and reduced mTORC1 in the decidua from RPL may participate in dNK cell dysfunction. In line with this, previous investigation had showed that obesity-driven changes to human dNK critically impair placental development (47). Although a recent study reported that metabolite concentrations including glucose, pyruvate and lactate from RPL decidua were not significantly changed compared with that of healthy controls (48). This may arise from individual differences, as study have revealed that RPL is a heterogeneous disease (49). Indeed, another proteomics analysis on the RPL decidua reported an abnormal mitochondrial oxidative stress (50).

This study was the first to focus on the potential roles of different cellular metabolic processes in dNK function and the underlying mechanism. However, this study has several limitations. First and foremost, this work is limited by the cell-based experiment treated with pharmacological inhibitors *in vitro*. When used at high concentrations, the off-target effects of inhibitors may exist on NK cell metabolism, which warrants further elegant investigations using genetic models. Secondly, we used DICs instead of sorted dNK in some experiments. Other immune cells existing in DICs may confound the results in this scenario. The use of purified dNK probably would have been more appropriate, and should the same effects after metabolism alteration is found, our conclusions would have been strengthened. However, using DICs can take cross-talk between leukocytes into consideration, more like physiological conditions do. At last, decidual tissue of RPL was obtained after fetal demise. This brings the question of *“chicken or egg”* problem, which is hard to answer. Further study obtaining tissues of peri-implantation endometrium from patients with a history of RPL may give us more information.

In conclusion, this study highlights dNK metabolism profile and their regulatory effects and underlines the potential role of mTORC1 in dNK metabolism and function under physiological and pathological conditions. At present, the evidence that metabolism controls cell functional fate in health and diseases has made the strategy of targeting metabolism therapeutically possible. Given the results highlighted in this study, it is conceivable that impaired dNK metabolism may impact dNK cell function. Hence, targeting metabolism action could provide a new therapeutic strategy for pregnancy-related diseases, including RPL.

## Data Availability Statement

The original contributions presented in the study are included in the article/**Supplementary Material**. Further inquiries can be directed to the corresponding author.

## Ethics Statement

The studies involving human participants were reviewed and approved by the Ethics Committee of Tangdu Hospital, Air Force Medical University. The patients/participants provided their written informed consent to participate in this study.

## Author Contributions

XW, JD, and SY designed the study. SY, CQ, QX, and HL performed the experimental work. SY and JD wrote the manuscript. SY and SC performed the statistical analysis. XW, JD, SY, and SC prepared and revised the article. All authors gave final approval. XW was responsible for the supervision and project administration. All authors discussed, edited, and approved the final version.

## Funding

This work was supported by the National Natural Science Foundation of China (Grant No. 81871182), and the Platform Construction Fund of Tangdu Hospital (2020XKPT003).

## Conflict of Interest

The authors declare that the research was conducted in the absence of any commercial or financial relationships that could be construed as a potential conflict of interest.

## Publisher’s Note

All claims expressed in this article are solely those of the authors and do not necessarily represent those of their affiliated organizations, or those of the publisher, the editors and the reviewers. Any product that may be evaluated in this article, or claim that may be made by its manufacturer, is not guaranteed or endorsed by the publisher.
