# Supplementary material for: The mTORC1 Signaling Support Cellular Metabolism to Dictate Decidual NK Cells Function in Early Pregnancy
Source: Front Immunol. 2022 Mar 10;13:771732. doi: 10.3389/fimmu.2022.771732 (PMC8960317; doi:10.3389/fimmu.2022.771732)
Supplement: Supplementary file 1 [file DataSheet_1.docx]

Supplementary Material

The mTORC1 Signaling Support Cellular Metabolism to Dictate Decidual NK Cells Function in Early Pregnancy

# Supplementary Figures and Tables

## Supplementary Figures


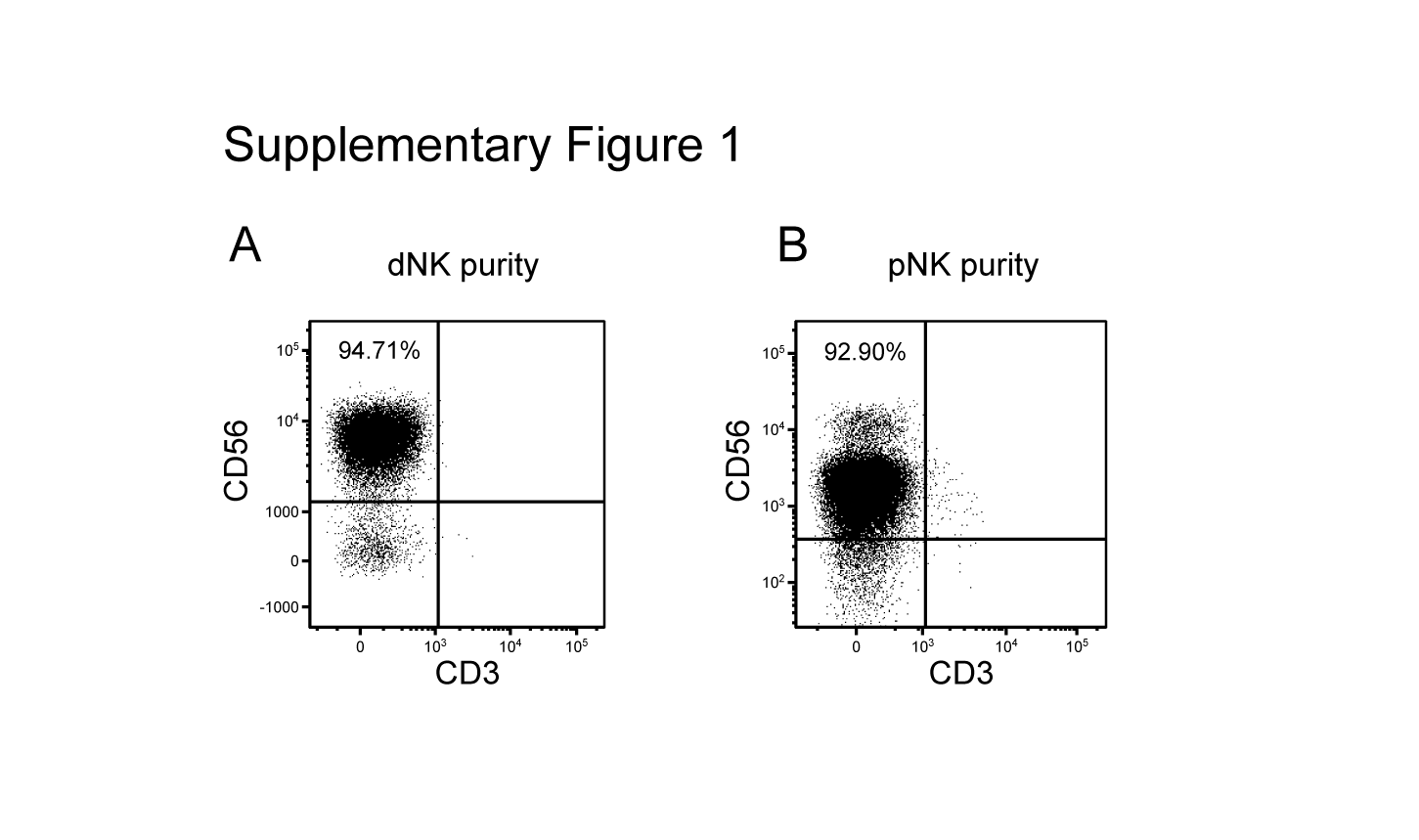


**Supplementary Figure 1.** **Purity of enriched human decidual NK cells (dNK) and peripheral NK cells (pNK).** (A, B) Representative flow cytometry plots of gated CD3^-^CD56^+^ NK cells.

## Supplementary Tables

**Supplementary Table 1. Antibodies for flow cytometry.**

| **Antibody** | **Catalogue Number** | **Source** |
| --- | --- | --- |
| APC/Cyanine7 anti-human CD3 | 300426 | Biolegend |
| PE anti-human CD56 (NCAM) | 318306 | Biolegend |
| FITC anti-human CD45 | 304005 | Biolegend |
| APC anti-human CD56 (NCAM) | 318310 | Biolegend |
| PerCP/Cyanine5.5 anti-human IFN-γ | 502525 | Biolegend |
| PE/Cyanine7 anti-human TNF-α | 502929 | Biolegend |
| PE anti-mouse/human Ki-67 | 151209 | Biolegend |
| FITC anti-human CD3 | 300306 | Biolegend |
| APC anti-human CD107a | 328620 | Biolegend |
| APC/Cyanine7 Mouse IgG1, κ Isotype Ctrl | 400127 | Biolegend |
| PE Mouse IgG1, κ Isotype Ctrl | 400113 | Biolegend |
| APC Mouse IgG1, κ Isotype Ctrl | 400121 | Biolegend |
| PerCP/Cyanine5.5 Mouse IgG1, κ Isotype Ctrl | 400149 | Biolegend |
| PE/Cyanine7 Mouse IgG1, κ Isotype Ctrl | 400125 | Biolegend |
| PE Rat IgG2b, κ Isotype Ctrl | 400607 | Biolegend |
| FITC Mouse IgG2a, κ Isotype Ctrl | 400207 | Biolegend |
| Human VEGF PE-conjugated Antibody | IC2931P | R&D Systems |
